# Therapeutic Electricity and Practical Muscle Testing

**Published:** 1900-03

**Authors:** 


**Therapeutic Electricity and Practical Muscle Testing.** By W. S. Hedley, M.D. Pp. ix., 278. London: J. & A. Churchill. 1899.

Strictly speaking, the term "therapeutic electricity" includes only the direct application of currents to the living body. The value of this book is, however, greatly increased by the author's wise decision to add a description of the electric cautery and the electric illumination of cavities.

The work is divided into three parts. The first deals briefly with general principles—the definition of "pressure" and "potential," the statement and applications of Ohm's law, the measurement of E.M.F., and so forth. Incidentally, it is pointed out that the choice and arrangement of cells has important practical bearings. Dr. Hedley insists that the rapidity of vibration of induced currents should be variable at will; and the absence of provision for this requirement is a defect of most English and German coils. The equipment for "static" treatment is figured and described in detail; and a chapter is devoted to the currents of great frequency and high potential first investigated by d'Arsonval, accredited with remarkable nutritive effects, and employed with considerable success in some cases of diabetes. The first part concludes with a chapter on the use of "Current from the Main." In the second, the electro-therapeutic outfit is described; the physiological effects of electricity are discussed under such headings as "electrolysis," "cataphoresis," and "electrotonus"; the technique of electro-diagnosis is explained; and the motor points are indicated by drawings after Castex. In the section which explains the normal law of contraction, the text would, we think, be rendered somewhat clearer by the addition to Fig. 68, which illustrates the position of the "virtual" anode and kathode when the actual anode is applied over a nerve, a second diagram illustrating their positions when the *kathode* is so applied. To most readers the third section of the book will be the most attractive. Here the various modes of general and local electrization are described, and the average "dosage" is, wherever possible (but only approximately), indicated. The electro-therapeutics of numerous diseases is then given at some length, with a few illustrative cases. Thus the author describes a case of ataxic paraplegia treated by himself with truly remarkable and lasting success by galvanisation of the cord and hydro-electric baths. There is also an account of a case of trigeminal neuralgia relieved by Bailey by the cataphoretic introduction of cocaine and aconite. To the fascinating subject of cataphoretic medication a chapter is devoted. The author has some interesting remarks on the electrical treatment of ileus and *post-partum* hemorrhage.

We cordially recommend this book as a thoroughly conscientious piece of work. A brief but comprehensive manual of electro-therapeutics was certainly wanted, and Dr. Hedley's book seems to us to meet every reasonable requirement. It certainly does not err on the side of over-statement: there is no unscientific exaggeration of the scope of electrical treatment. The book is interesting throughout, though in places it is rather stiff reading. We note two or three misprints: on page 168, "kalelectrotonus"; on page 171, "Hodge" (? Head); on p. 253, "guteal," for gluteal.

